# Differences in structural parameters in patients with open-angle glaucoma, high myopia and both diseases concurrently. A pilot study

**DOI:** 10.1371/journal.pone.0286019

**Published:** 2023-06-22

**Authors:** Agne Markeviciute, Ingrida Januleviciene, Gal Antman, Brent Siesky, Alon Harris

**Affiliations:** 1 Department of Ophthalmology, Medical Academy, Lithuanian University of Health Sciences, Kaunas, Lithuania; 2 Department of Ophthalmology, Icahn School of Medicine at Mount Sinai, New York, NY, United States of America; 3 Department of Ophthalmology, Rabin Medical Center, Petach Tikwa, Israel; Bascom Palmer Eye Institute, UNITED STATES

## Abstract

**Purpose:**

To evaluate the differences in structural parameters in patients with open-angle glaucoma (OAG), high myopia (M), and both diseases (OAG-M) concurrently.

**Methods:**

42 subjects with OAG (n = 14), M (n = 14) and OAG-M (n = 14) were included in a prospective pilot study. Mean peripapillary retinal nerve fiber layer (RNFL) thickness, RNFL in superior, temporal, inferior, nasal quadrants, macular ganglion cell complex (GCC) and its’ layers, vessel density (VD) of optic nerve head (ONH) and macula were evaluated.

**Results:**

The OAG-M group showed significantly lowest thickness of mean peripapillary RNFL 89 (49–103) μm (p = 0.021), temporal quadrant 64.5 (51–109) μm (p = 0.001) and inferior quadrant 107 (64–124) μm (p = 0.025). The macular RNFL was thinnest in the OAG-M group (p <0.001). Macular VD in inferior quadrant was lowest in OAG-M group at superficial capillary plexus 45.92 (40.39–51.72) % (p = 0.014) and choriocapillaris 51.62 (49.87–56.63) % (p = 0.035). The lowest ONH VD of temporal quadrant was found in the OAG-M group 52.15 (35.73–59.53) % (p = 0.001) in the superficial capillary plexus. Similarly, the lowest VD of inferior quadrant was found in OAG-M group in the choriocapillaris 54.42 (46.31–64.64) % (p<0.001).

**Conclusions:**

The M group showed the least thinning in the peripapillary RNFL thickness in the temporal quadrant and macular RNFL compared to other two groups. The highest macular VD in the inferior quadrant was in the M group in the superficial capillary plexus, deep capillary plexus and choriocapillaris. The M group showed highest VD in the temporal quadrant and in total VD of ONH at the superficial capillary plexus and in total VD of ONH at the deep capillary plexus.

**Practical recommendations:**

The observed decrease in peripapillary RNFL thickness of the temporal quadrant, macular RNFL thickness, the decrease of macular VD at the inferior quadrant and decrease in VD of the ONH temporal quadrant in deep capillary plexus could be beneficial for diagnosing glaucoma in high myopia.

## Introduction

Myopia is a growing public health problem predicted to affect 5 billion people worldwide (~50% of the world population), while high myopia is expected to affect 9.8% of the world population by 2050 [1]. The impact of myopia is broad and highly significant, yet the definitions used to grade myopia varies between studies and is often without consensus. For instance, Holden et al. performed a systematic review and found that 30.5% of studies defined and measured high myopia as −6.00 diopters (D) or less, 35.6% defined it as −5.00 D or less, 1.7% defined it as −8.00 D or less, and 1.7% defined it as −3.00 D or less [1]. Better understanding of myopia thresholds is important as high myopia is a significant risk factor for glaucoma, especially in certain ethnic groups [2].

Primary open-angle glaucoma (OAG) is defined as a chronic, progressive, and irreversible multifactorial optic neuropathy that is characterized by an open angle of the anterior chamber, optic nerve head changes, and progressive visual field loss [3]. Understanding refractive error is important in determining risk for OAG as it has been reported that patients with high myopia were six times more likely to develop glaucoma [4]. As with myopia, POAG is a disease growing in prevalence affecting approximately 111.8 million people worldwide by 2040 [5]. The increase in the prevalence of both diseases combined with the association between POAG and myopia demonstrate the need for improved understanding of both conditions.

The prevalence of POAG increases with age, elevated intraocular pressure (IOP), myopia and increasing ocular axial length (AL) [6]. While myopia has been shown to be a risk factor for OAG, the exact mechanisms underlying the association between myopia and the development of glaucoma has been discussed, but not established [7–10]. Specifically, it has been reported that the structural changes of optic nerve head (ONH) that occur in myopia might induce the onset and progression of glaucomatous changes [7]. This may be due to the fact that as the length of the ocular axis increases, the sclera stretches oppositely to the choroid and retina; thus, atrophy of the choroid and retinal pigment epithelium develops [8]. In conjunction with a longer ocular AL, the lamina cribrosa and the peripapillary edge of the sclera become stretched and thinner; thus, the mechanical support of the lamina cribrosa is lost, making optic nerve fibres passing through the lamina cribrosa more susceptible to IOP changes [7,9]. Furthermore, the tilt and torsion of the ONH can cause direct mechanical stress to the axons and an indirect reduction in the structural and functional support of the axons due to the deformation of lamina cribrosa [7]. However, it was emphasized that these structural changes may induce glaucomatous changes only in the presence of increased IOP [10].

Diagnosis of glaucoma is based on the structural changes of ONH and retinal nerve fiber layer (RNFL), and corresponding visual field defects. Decreases in peripapillary RNFL thickness, macular thickness, and ganglion cell complex (GCC) are all also observed with glaucoma progression [11]. In addition, all parts of the macular ganglion cell complex, including ganglion cell dendrites (inner plexiform layer), ganglion cell bodies (ganglion cell layer), and ganglion cell axons (RNFL) are affected in glaucoma [12]. Previously it was reported that the assessment of the thickness of macular GCC and its layers is at least equally accurate for diagnosing glaucoma compared to the evaluation of the thickness of peripapillary RNFL [13,14]. It has been suggested that changes in the thickness of GCC may occur even earlier than the changes of peripapillary RNFL in glaucoma [15]. In healthy eyes, ganglion cell bodies and axons are distributed symmetrically between the upper and lower retinal hemispheres, and glaucoma changes are often asymmetric [16].

Patients with high myopia may be over-diagnosed with glaucoma as evaluating possible glaucomatous changes in highly myopic eyes involves an obstructed oblique view of the ONH, increased pallor of the neuroretinal rim, and flattening of the cup [7]. Posterior staphylomas and macular or chorioretinal atrophy may also lead to visual field defects [7,17]. Lee et al. found that 16.1% of healthy, young patients with high myopia had typical glaucomatous visual field defects, and 25.6% had a widespread blind spot [18]. These data point to the need to better elucidate myopia levels in OAG to understand the potential shared risk and improve diagnostic specificity for both conditions.

It is also important to note that structural changes associated with high myopia may lead to misinterpretation of imaging data and the results of studies that evaluated changes in the RNFL thickness and its association with myopia are controversial. Lee et al. reported that RNFL thickness decreased significantly more in high myopia during two years of study, with more significant changes among older subjects [19]. Zha and colleagues found that myopic eyes had a thinner average global RNFL thickness compared with emmetropic eyes, however, it was not associated with age or gender [20]. It was also proposed that RNFL thickness has a linear increase with the increase of spherical equivalent (SE) [20]. Furthermore, it is known that the thickness of RNFL decreases by about 0.2–0.5 μm per year in healthy subjects [19,21] and the thickness of the macular ganglion cell-inner plexiform layer (GCL+) decreases by about 0.31 μm per year [22].

Increased ocular AL has also been associated with reduced RNFL thickness in myopia. Previously Budenz et al. found that for every 1-mm-greater ocular AL, mean RNFL thickness decreased by approximately 2.2 μm [23]. The superotemporal and inferotemporal RNFL bundles in myopia tend to get closer together temporally due to the longer AL and elongation of the fovea-optic disc distance [24]. Seo et al. showed that the thickness of peripapillary RNFL decreased respectively with the increasing degree of myopia, except for the temporal quadrant in which thickening of RNFL was observed [25].

Assessment of macular GCC may overcome the difficulties of assessing peripapillary ONH RNFL thickness in highly myopic eyes. Several studies did not find significant associations between the decrease of GCC thickness and AL in myopia, thus suggesting that the thickness of macular GCC and its layers may be less affected by changes in AL or refractive errors than peripapillary RNFL thickness [26–29]. Furthermore, the assessment of GCC thickness had significantly better results in detecting glaucomatous changes in high myopia than the assessment of peripapillary RNFL thickness [30,31]. Longer AL in myopia and mechanical stretching of the retina may result in a lower GCC density over a larger surface area of the retina [25,32] thus leading to the false-positive decrease of macular GCC thickness.

Applications of optical coherence tomography angiography (OCTA) allow for the non-invasive evaluation of microvascular changes in the retinal and choroidal layers. Previously it was found that vessel density (VD) decreased significantly in the peripapillary and perifoveal areas during glaucoma, and decreased VD was associated with the severity of glaucoma as well as with visual field defects [33]. Increased AL and thinning of retinal tissue in myopia may reduce oxygen requirements leading to decreased blood circulation, impaired retinal and choroidal blood flow [34–37]. As these data are controversial, our pilot study aimed to evaluate and compare the differences in ocular structural parameters in patients with OAG, high myopia (M), and both diseases (OAG-M) concurrently.

## Methods

Forty-two Caucasian subjects with OAG (n = 14), high myopia (M) (n = 14), and both diseases (OAG-M) (n = 14) were included in a prospective pilot study. All study procedures were conducted at the Lithuanian University of Health Sciences (LUHS) Kaunas Clinics Eye Clinic. The Kaunas Regional Biomedical Research Ethics Committee approved the study protocol and all participants provided written informed consent, with procedures conforming to the Declaration of Helsinki. Both eyes were examined with one random (coin flip) eye per patient included in the study.

Inclusion criteria were: age between 30–65 years, no objective findings of macular diseases (confirmed by the researcher), confirmed diagnosis of OAG by a general ophthalmologist, RNFL defects corresponding to visual field defects with mean deviation (MD) ≥-12 dB and high myopia with spherical equivalent (SE) ≤ −6.0 D.

Measurements included: best-corrected visual acuity (BCVA), Schiotz tonometry, autorefractometry, standard automated perimetry (Humphrey 24–2 SITA Standard), OCT and OCT angiography (Swept source DRI-OCT Triton. Topcon. Tokyo; Japan). Volumetric optic disc OCT scans were acquired from a 6 × 6 mm cube. NRA–neuroretinal rim area (mm^2^), DA–disc area (mm^2^), linear cup/disc ratio, vertical cup/disc ratio, mean RNFL thickness (μm), RNFL thickness in superior (S), nasal (N), inferior (I) and temporal (T) quadrants were measured. Volumetric macular scans were acquired from a 9 x 9 mm cube. RNFL thickness (μm), ganglion cell-inner plexiform layer (GCL+) thickness and ganglion cell complex (GCC including RNFL and GCL+ layers) thickness were assessed. The difference between the higher and lower values of superior and inferior hemispheres of GCL+ and GCC thickness was calculated.

The OCTA scans were performed using volumetric scans covering an area of 4.5 × 4.5 mm centered around the optic disc and 6x6 mm around the macula. OCTA software performed automated segmentation of the retinal layers and calculated macular vascular density (VD %) automatically. We assessed VD in S, N, I, and T quadrants in four layers: superficial capillary plexus, deep capillary plexus, outer retinal capillary plexus, and the choriocapillaris.

**Fig 1:**
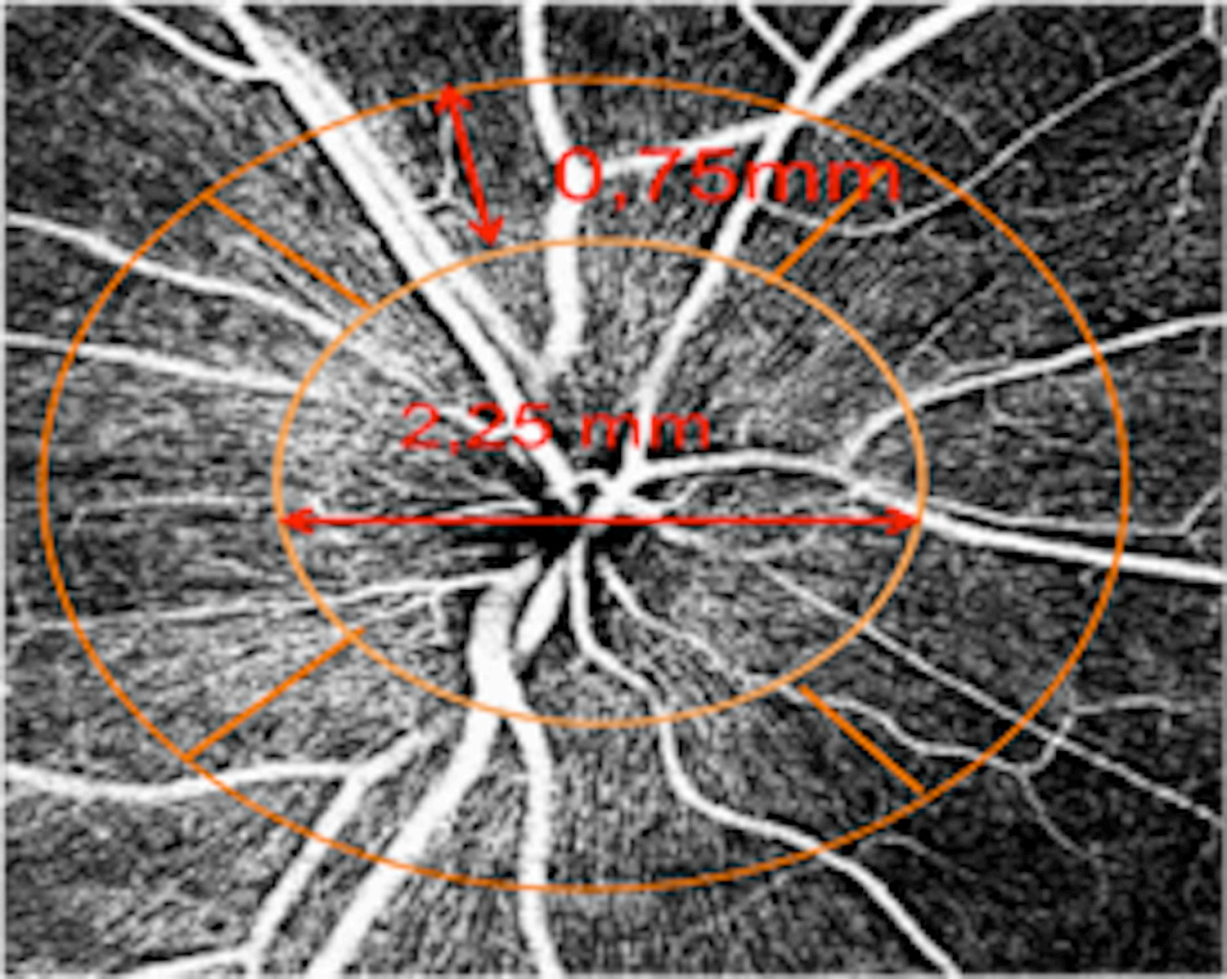
Optic nerve head grid used for vessel density measurement.

Automated segmentation of the OCTA data was used to get en-face ONH OCTA images in the superficial capillary plexus, deep capillary plexus and choriocapillaris. We used a manual grid (Fig 1) modified by the Garway-Heath map and divided it into S, I, N and T sectors. Each superior and inferior sector covered 80°, temporal 90°, and nasal 110°. The grid was centered on OCTA *en face* images. The VD was measured and calculated using ImageJ v 1.53a software (National Institute of Health. USA). The size of each image was 320 x 320 pixels, and we used the threshold Phansalkar for binarization. The ONH VD was calculated as the percentage of the sampled area occupied by vessels detected by the thresholding parameters. The total VD of ONH and VD of S, I, N, and T quadrants were measured.

Statistical analysis was performed using the computer program SPSS 25.0. Methods of descriptive statistics defined all variables. The analysis of the quantitative variables included the calculation of the mean and standard deviation (SD). The chi-square test was used to determine the interdependence between qualitative variables (χ2).

The hypothesis of equality among three groups was analyzed using the Kruskal-Wallis test. The results of nonparametric tests were described as median, minimum and maximum values. A pairwise comparison was used to assess the significant differences between groups. Spearman’s correlation assessed the association between categorical or abnormally distributed continuous variables. P < 0.05 was considered significant.

## Results

### Demographics

Forty-two patients (71.43% women; 28.57% men) with OAG, high myopia (M), OAG and high myopia (OAG-M) were included in the study, each group consisting of 14 subjects. No statistically significant difference was found between sex and age (p> 0.05) in the groups (Table 1).

**Table 1:** Demographic characteristics of study subjects.

		OAG (n = 14) Mean (SD)	M (n = 14) Mean (SD)	OAG-M (n = 14) Mean (SD)	p level
Sex	Female n (%)	11 (78.6)	12 (85.7)	7 (50)	0.086
	Male n (%)	3 (21.4)	2 (14.3)	7 (50)	
Age (years)		54.7 (0.8)	52.0 (0.8)	50.5 (2.6)	0.201

OAG—open-angle glaucoma; M—high myopia; OAG-M -open-angle glaucoma and high myopia; n -number; SD—standard deviation; p—significance level, p<0.05.

### Clinical characteristics and ONH structural parameters

Changes in SE, BCVA, IOP and ONH parameters are shown in Table 2. BCVA did not differ significantly between the groups. No significant difference in SE values was found between the M and OAG-M groups (after pairwise comparison p = 0.449). Furthermore, no significant difference in IOP values between the OAG and OAG-M groups (p = 0.431) was found. Neuroretinal rim area (NRA) was found to be significantly lowest at 0.9 (0.1–1.9) mm^2^ in OAG group. Furthermore, it was significantly lower in OAG-M group when compared to M group (after pairwise comparison p = 0.027). Vertical and horizontal cup to disc ratios were significantly highest in OAG patients (p<0.05).

**Table 2:** Clinical characteristics in OAG, M, OAG-M groups.

	OAG (n = 14) Median (min-max)	M (n = 14) Median (min-max)	OAG-M (n = 14) Median (min-max)	p level
SE (D)	0.12 (-0.5; +1.5)	-7.25 (-9.25; -6.0)	-6.5 (-12.0; -6.0)	**<0.001**
BCVA (Snellen Decimal)	1.0 (0.9–1.0)	1.0 (0.8–1.0)	1.0 (0.6–1.0)	0.135
IOP (mmHg)	13.4 (10.2–17.3)	12.9 (10.2–16.2)	15.3 (11.2–17.3)	0.06
NRA (mm^2^)	0.9 (0.1–1.9)	1.6 (1.3–2.4)	1.1 (0.6–2.2)	**0.003**
DA (mm^2^)	2.0 (1.5–2.5)	1.9 (1.4–2.5)	2.1 (1.1–3.1)	0.670
Vertical cup-disc ratio	0.7 (0.4–1.0)	0.4 (0–0.5)	0.5 (0–0.9)	**0.001**
Horizontal cup-disc ratio	0.7 (0.4–1.0)	0.3 (0–0.5)	0.4 (0–0.8)	**<0.001**

OAG—open-angle glaucoma; M—high myopia; OAG-M—open-angle glaucoma and high myopia; n -number; SE—spherical equivalent; Min—the lowest value; Max—the highest level; BCVA- best-corrected visual acuity; NRA—neuroretinal rim area; DA—disc area; p—significance level, p<0.05.

The differences in peripapillary RNFL, macular RNFL, GCL+, and GCC thickness are presented in Table 3. The statistically significant lowest thickness of mean RNFL 89 (49–103) μm, RNFL thickness of T quadrant 64.5 (51–109) μm, and I quadrant 107 (64–124) μm were found in OAG-M patients. RNFL thickness of the T quadrant was found to be significantly highest in the M group compared to the OAG group (p = 0.009) and the OAG-M group (p = 0.003).

**Table 3:** Peripapillary RNFL and macular RNFL, GCL+, GCC.

		OAG (n = 14) Median (min-max)	M (n = 14) Median (min-max)	OAG-M (n = 14) Median (min-max)	p
**Peripapillary**	Mean RNFL (μm)	99 (62–117)	97 (86–106)	89 (49–103)	**0.021**
	RNFL S (μm)	110 (81–148)	106.5 (20–143)	117 (101–140)	0.150
	RNFL T (μm)	71.5 (45–94)	83 (72–153)	64.5 (51–109)	**0.001**
	RNFL I (μm)	118.4 (70–144)	110 (99–132)	107 (64–124)	**0.025**
	RNFL N (μm)	83 (45–99)	67 (44–86)	69.5 (32–86)	0.055
**Macula**	RNFL (μm)	40 (25–42)	44 (41–52)	38.5 (25–48)	**<0.001**
	GCL+ (μm)	62 (52–66)	58.5 (56–62)	56 (46–63)	0.342
	GCC (μm)	100 (78–108)	102 (97–109)	93.5 (72–112)	0.129
	GCL+ difference	2 (0–7)	1.0 (0–5)	3.5 (0–9)	**0.025**
	GCC difference	5 (1–22)	4.5 (1–11)	2 (1–22)	0.675

OAG—open-angle glaucoma; M—high myopia; OAG-M -open angle glaucoma and high myopia; n -number; Min—the lowest value; Max—the highest level; RNFL—retinal nerve fiber layer; GCL+—ganglion cell-inner plexiform layer; GCC—ganglion cell complex; S-superior; I—inferior; N—nasal; T—temporal; p—significance level, p<0.05.

Only macular RNFL thickness was significantly different being significantly lower in the OAG group than in the M group (p = 0.001) and significantly lower in OAG-M than in the M group (p = 0.028). The difference between the GCL+ thickness in the upper and lower segments was highest in the OAG-M group, when compared to OAG (p = 0.017) and OAG-M (p = 0.027).

### Macular and ONH OCT angiography

Statistically significant results of macular and ONH OCTA are shown in Table 4. Specifically, the lowest VD of I quadrant 45.92 (40.39–51.72)%, was found in OAG-M at superficial capillary plexus and in the choriocapillaris 51.62 (49.87–56.63)%. In the deep capillary plexus, VD was significantly lowest in the OAG group, 47.96 (44.68–53.5)%. ONH OCTA results were evaluated in previously discussed sectors. In the superficial capillary plexus, the VD of the T quadrant was significantly lower in OAG than in M subjects (p = 0.006) and lower in OAG-M than in M subjects (p = 0.002). The VD of the total ONH area was significantly lowest in OAG patients compared to M (p = 0.017) and OAG-M (p = 0.02). Patients with OAG had the lowest VD in in the T quadrant 54.64 (51.22–62.12)% (p = 0.006) and total ONH VD 50.65 (41.44–54.34)% (p = 0.006) in deep capillary plexus. OAG-M subjects had the lowest VD in the I quadrant in the choriocapillaris layer (p<0.001).

**Table 4:** ONH and macular OCTA results.

		OAG (n = 14) Median (min-max)	M (n = 14) Median (min-max)	OAG-M (n = 14) Median (min-max)	p
**Macula** (VD%)	**Superficial capillary plexus**				
	I	46.84 (40.34–50.52)	50.98 (43.25–54.99)	45.92 (40.39–51.72)	**0.014**
	**Deep capillary plexus**				
	I	47.96 (44.68–53.5)	52.77 (42.75–64.16)	50.89 (44.53–56.49)	**0.013**
	**Choriocapillaris**				
	I	52.31 (49.28–55.92)	54.17 (49.26–55.53)	51.62 (49.87–56.63)	**0.035**
**ONH** (VD%)	**Superficial capillary plexus**				
	T	53.16 (45.13–60.25)	57.62 (52.47–63.98)	52.15 (35.73–59.53)	**0.001**
	Total	50.97 (44.49–57.47)	54.31 (50.61–56.81)	51.14 (42.85–55.15)	**0.006**
	**Deep capillary plexus**				
	T	54.64 (51.22–62.12)	58.38 (53.70–65.18)	57.19 (53.48–58.79)	**0.006**
	Total	50.65 (41.44–54.34)	53.95 (48.62–56.04)	52.06 (41.43–55.53)	**0.006**
	**Choriocapillaris**				
	I	60.67 (54.66–72.62)	64.29 (58.85–72.8)	54.42 (46.31–64.64)	**<0.001**

OAG—open angle glaucoma; M—high myopia; OAG-M -open angle glaucoma and high myopia; n -number; Min—the lowest value; Max—the highest level; RNFL—retinal nerve fiber layer; ONH—optic nerve head; OCTA—optical coherence tomography angiography; VD—vessel density; I—inferior; T -temporal; p—significance level, p<0.05.

Correlations between mean peripapillary RNFL thickness and each of the macular layers: RNFL, GCL +, and GCC thickness were assessed in OAG, M, and OAG-M groups separately. Significant positive correlations were found in all groups, except for the M group. No significant correlation was found between the peripapillary RNFL and macular RNFL thickness (r = 0.384; p = 0.175). The strongest correlation was found between the mean peripapillary RNFL and GCC thickness. Results of all group correlations are presented in Table 5.

**Table 5:** Correlations between the mean peripapillary RNFL thickness and macular parameters.

		OAG		OAG-M		M	
		r	p	r	p	r	p
Peripapillary RNFL	mRNFL	0.707	**0.007**	0.823	**0.003**	0.384	0.175
	mGCL+	0.674	**0.008**	0.583	**0.029**	0.661	**0.01**
	mGCC	0.764	**0.001**	0.862	**<0.001**	0.718	**0.004**

OAG—open angle glaucoma; M—high myopia; OAG-M -open angle glaucoma and high myopia; RNFL—retinal nerve fiber layer; m -macular; GCL+—ganglion cell-inner plexiform layer; GCC—ganglion cell complex; r—Spearman’s correlation coefficient; p—significance level, p<0.05.

Correlations between mean peripapillary RNFL thickness and ONH VD and between macular GCL+ thickness and ONH VD were evaluated in OAG and M groups. Strong positive correlations were found between the mean peripapillary RNFL thickness and VD in the T quadrant as well as with the total ONH VD at deep capillary plexus in the OAG group. Contrary, a significant moderate negative correlation was found in the M group. Significant correlations are shown in Table 6.

**Table 6:** Correlations between peripapillary RNFL and ONH VD in the OAG and M groups.

	OAG			M
Peripapillary RNFL	III	T (VD)	Total (VD)	T (VD)
		**r = 0.781** **p = 0.001**	**r = 0.734** **p = 0.004**	**r = -0.606** **p = 0.022**

OAG—open angle glaucoma; M—high myopia; RNFL—retinal nerve fiber layer; III—deep capillary plexus; T—temporal; VD—vessel density; r—Spearman’s correlation coefficient; p—significance level, p<0.05.

A significant moderate positive correlation was found between macular GCL+ thickness and ONH VD of the T quadrant in the deep capillary plexus in OAG patients. Conversely, a significant negative correlations were found between macular GCL+ thickness and VD of ONH in all quadrants except for the S (r = −0.377; p = 0.184) in M group. We evaluated the associations between macular GCL+ thickness and ONH VD of all the quadrants in three different layers. However, we present only significant results in Table 7.

**Table 7:** Correlations between macular GCL+ and ONH VD in OAG and M groups.

mGCL+		**OAG**	**M**			
	III	T (VD)	T(VD)	I(VD)	N(VD)	Total (VD)
		**r = 0.693** **p = 0.006**	**r = -0.638** **p = 0.014**	**r = -0.703** **p = 0.005**	**r = -0.698** **p = 0.005**	**r = -0.691** **p = 0.009**

OAG—open angle glaucoma; M—high myopia; m- macular; GCL+—ganglion cell-inner plexiform layer; T -temporal; I—inferior; N—nasal; VD—vessel density; III—deep capillary plexus; r—Spearman’s correlation coefficient, p<0.05.

## Discussion

This pilot study assessed differences in structural parameters that are often used to distinguish glaucomatous changes from those associated with high myopia. In our analysis, we found that the NRA was significantly higher in patients with high myopia and significantly lower in patients with OAG and high myopia, when compared to high myopia subjects. Although it has been suggested that assessment of NRA may not be accurate in high myopia, Kim et al. found that NRA demonstrated better accuracy for glaucoma detection in myopic eyes compared to peripapillary RNFL thickness [38]. Similarly, Aref and colleagues reported that the false positive rate of glaucomatous changes in highly myopic eyes was significantly higher using peripapillary RNFL and macular GCL+ parameters when compared to ONH NRA and cup to disc ratio [39]. Furthermore, several studies found no significant association between NRA and optic disc changes and ocular AL or SE [32,40,41]. Taken together, we hypothesize that NRA may be one of the target features available for diagnosing glaucoma in patients with high myopia.

In addition we found that peripapillary RNFL thickness in the T quadrant was significantly higher in patients with high myopia when compared with OAG and OAG-M groups. This corresponds with other studies’ results that observed thickening of RNFL in the T quadrant in high myopia due to the temporalization of RNFL due to increased AL [25,42–44]. The RNFL thickness in the T quadrant is expected to be the least affected by high myopia changes suggesting that a decrease in RNFL thickness in this part could indicate glaucomatous changes [44]. Previously, Tai and colleagues found that mean and I quadrant RNFL thickness was lower in high myopia than in OAG patients [45]. Shoji et al. also reported that as the degree of myopia increased, the thickness of RNFL decreased the most in the I quadrant, followed by the S quadrant [28]. These areas were reported to be structurally weakest parts of lamina cribrosa, thus making it more vulnerable to changes in glaucoma and high myopia [46]. In our study, the mean thickness of peripapillary RNFL and thickness in I, N, and S quadrants were lower in patients with high myopia than in those with OAG, however, the results were not statistically significant. Therefore, these data suggest that evaluating mean RNFL and RNFL thickness in the I, S, and N quadrants may lead to an increase in false-positive glaucomatous changes in high myopia.

Our results also showed that mean peripapillary RNFL thickness correlated positively with all the evaluated macular structural parameters. The strongest association was found between mean peripapillary RNFL thickness and macular GCC thickness in OAG and OAG-M groups. These data suggest glaucomatous changes in persons with significant myopia may be best determined in part by evaluating the thickness of macular GCC and its layers. This is due to the fact that we found that only macular RNFL thickness was significantly lower in OAG-M patients compared with M patients. This suggests that the decrease of macular RNFL thickness may be advantageous for diagnosing glaucoma in patients with high myopia.

Although we did not find statistically significant differences in GCL+ thickness between the study groups, we observed that GCL+ thickness was lower in high myopia than in OAG patients. This is consistent with the results of a study by Seo et al. in which they found that the thickness of GCL+ decreased with increasing AL in high myopia [25]. Choi et al. also reported that GCL+ thickness was significantly lower in high myopia [32]. This suggests that observations of GCL+ thickness alone may lead to erroneous results for diagnosing glaucoma in high myopia.

Previously, Wang et al. reported that macular GCC thickness was significantly lower in patients with OAG and high myopia than in those with high myopia [42]. Although we found no statistically significant differences, GCC thickness was lower in the OAG-M group and highest in the M group. We also found the strongest correlation in biomarkers between mean peripapillary RNFL thickness and thickness of macular GCC thickness. However, Wang and colleagues observed that GCC thickness was significantly reduced in high myopia compared to emmetropia, suggesting that GCC thickness may be affected by high myopia [42]. Nevertheless, it was indicated that GCC had a higher diagnostic power in detecting glaucoma in high myopia than peripapillary RNFL thickness [28,42]. This highlights the potential importance of including GCC evaluation in diagnosing glaucoma for persons with high myopia.

In our pilot analysis, the difference between superior and inferior segments of macular GCL+ thickness was significantly lowest in patients with high myopia (significantly lower when compared to OAG and OAG-M groups). Asymmetry between GCL+ thickness in the upper and lower segments was suggested as an early sign of glaucoma in myopic patients; a difference of 5 μm is considered suspicious [15]. Although some authors reported that no significant decrease in macular VD was observed in patients with high myopia [36,47,48], others suggested that myopia may be associated with the decline in macular VD [49,50]. We found that macular VD in the I quadrant was significantly highest in patients with high myopia compared to OAG-M and OAG groups in superficial capillary plexus, deep capillary plexus, and choriocapillaris. In this group (M) of patients, myopia may have no or negligible effect on macular VD in the I quadrant, and a decrease in VD in this quadrant may be pathognomonic for glaucoma in high myopia. Similar results were reported by Lee et al. that evaluation of macular VD in the inferior quadrant had great diagnostic accuracy for glaucoma in patients with high myopia [36].

Although both are known to be important factors in OAG, the exact association between high myopia and changes in peripapillary VD has not been established. Some authors have suggested that peripapillary VD decreases with increasing ocular AL in high myopia [51,52]. However, others did not find significant associations [53]. In our analysis, we found that patients with high myopia had significantly highest total VD of ONH and VD in the T quadrant in superficial and deep capillary plexuses and VD in the I quadrant in the choriocapillaris. A strong positive correlation was found in the deep capillary plexus between VD of the T quadrant and mean peripapillary RNFL thickness in OAG patients; at the same time, a moderate negative correlation was found in M patients. This suggests that the temporal quadrant may be the least affected by high myopia as we did not find a decrease in VD. Similarly, Akagi et al. did not find a significant decrease in VD in the T quadrant in high myopia compared to emmetropia [54]. Thus changes in VD in the T quadrant in the deep capillary plexus may be a valuable parameter in diagnosing glaucoma in high myopia.

There is an important clinical need to identify a method to differentiate glaucomatous changes of ocular tissues from those observed in high myopia. Growing prevalence of both diseases and shared anatomical tissues points to the need for better understanding of the mechanism(s) of myopia that increase risk for OAG. Accessibility of examining tissues and differences in study methodologies in the literature including specific anatomical structures quantified and significantly different study groups currently make comparisons of data problematic. Currently, there is a lack of roust study data available and only a few studies evaluated similar study groups like ours (OAG, M, and OAG-M). Our pilot study results suggest that including assessment of macular GCC and OCTA results may improve specificity for diagnosis and management of OAG in patients with high myopia.

There are several limitations of our study to acknowledge. First our study is pilot in nature, with a relatively small sample size which may inflate or limit the results. High-quality outcomes could be obtained in future research studies with significantly larger sample sizes. In addition, we did not include and evaluate all physiological ocular parameters in patients, and including more features like AL may better help define the weight of all factors that influence structural parameters. Further, limited comparable data is available, definitions of myopia often vary in previous studies, and prospective data on OAG progression was not examined. Nevertheless, the results of our pilot analysis show the importance of identification of glaucoma-specific features in high myopia. Further prospective longitudinal studies with larger sample sizes are needed to better define glaucomatous progression changes from those associated only with high myopia.

### Practical recommendations

The observed decrease in peripapillary RNFL thickness of the T quadrant, macular RNFL thickness, the increase in the difference of macular GCL+ thickness between the upper and lower sectors, the decrease of macular VD at the I quadrant and decrease in VD of the ONH T quadrant in deep capillary plexus suggest that these biomarkers are beneficial for diagnosing glaucoma in high myopia.

## Supporting information

S1 Data (ZIP)
